# A “NEW” way to look at an “old” test: Transforming the void spot assay (VSA) into a diagnostic tool

**DOI:** 10.14814/phy2.14985

**Published:** 2021-08-02

**Authors:** Marianela G. Dalghi

**Affiliations:** ^1^ Renal‐Electrolyte Division Department of Medicine University of Pittsburgh Pittsburgh PA USA

Lower urinary tract (LUT) pathologies present in almost all cases with the clinical manifestations of altered voiding behavior, symptoms that include frequency, urgency, nocturia, incontinence, and dribbling. To study urinary pathologies using mouse models, as well as the efficacy of a given treatment or intervention, the voluntary voiding behavior of mice must be addressed. The void spot assay (VSA) is an increasingly common method used in the field that is simple, inexpensive, and noninvasive (Chen et al., 2017; Sugino et al., 2008). The first VSA ever performed—to the best of the author's knowledge—is the one reported by Desjardins et al. (1973), where the investigators studied the urination patterns of male mice according to their social rank. In this study, the investigators placed a filter paper on the floor of a mouse cage and examined the urine marks deposited using ultraviolet light after an overnight test. Since then, researchers have used this approach, with slight modifications, to assess the voiding behavior of mice under a wide variety of conditions and disease models (Chen et al., 2017; Keil et al., 2016; Liu et al., 2019; Sugino et al., 2008). Although technically simple, proper analysis and correct interpretation of the results are not trivial (Bjorling et al., 2015; Wegner et al., 2018). Most of the researchers in the field have focused their attention on parameters that include the total number of void spots in a specific time window (frequency of urination), the total size and the frequency distribution of the size of the void spots, and the region where the urine is deposited (corners *versus* center) which may be related to urinary continence, and compared these parameters between a control and a treated or disease group (Liu et al., 2019; Rajandram et al., 2016; Sugino et al., 2008).

The study by Ruetten et al. (2021) seeks to transform the VSA into a diagnostic tool. To this aim, the investigators created an approach to integrate all the output parameters of the VSA, that is, the number, the size distribution, and the region of the urine spots, to generate voiding patterns that allows them to classify mice based on their voiding dysfunction phenotypes. The investigators generated a protocol of analysis called Normalized Endpoint Work Through (NEW) to normalize the VSA endpoints of experimental mice to the control group, followed by principal component analysis and subsequent hierarchical clustering. The researchers analyzed the data obtained from male mice belonging to three different disease models—diabetic diuresis, bacteria‐induced prostatic inflammation, and hormone‐driven bladder outlet obstruction—and observed that each experimental group formed a fairly distinct cluster. The fact that each of the models tested presents with a particular cluster opens up the possibility to relate the VSA phenome to an etiology. Whether a cluster is unique to a given etiology or whether two conditions with similar manifestations but of different underlying cause can be resolved into separate clusters (e.g., different types of urinary incontinence) is yet to be determined. However, this study sets the basis for a novel way to analyze the results of the VSA and as more experimental data are generated representing wider varieties of LUT diseases, more clusters will emerge that may reveal interesting distinctions or overlaps, as shown by the authors when analyzing aged male mice. These mice exhibited a heterogeneous voiding behavior with some animals displaying a phenome typical of the diabetic diuresis or bladder outlet obstruction groups, while others formed a distinct cluster. These findings suggest that aged mice develop different types of LUT dysfunction symptoms. Future studies confirming that the aged mice that clustered in a certain group exhibit the biochemical (i.e., glycemia) or anatomical (i.e., prostate size) characteristics typical of that group will contribute to validate this approach.

One of the most challenging aspects of the VSA when performed as an endpoint test is the analysis of overlapping urine spots and the distinction between small voluntary void spots and deposits that result from carryover associated with the tail and paws. Another limitation is the temporal resolution that leads, for example, to the inability to assess whether a small void spot constitutes a voiding event by itself or is the dribbling product after other, larger spot. Then, combining this new approach of analysis to real‐time recording of the voiding events using methods such as video monitoring (Hou et al., 2016; Verstegen et al., 2020) should be considered in future studies as it will help with the unambiguous interpretation of the results at the time that it will contribute to additional relevant variables for building clusters.

Overall, Ruetten et al. (2021) described an exciting “NEW” way to look at an “old” test. This, together with the possibility of integrating anatomical, physiological, and behavioral findings to the VSA output parameters to build clusters, will greatly contribute to generate diagnostic tools for assessing LUT pathologies in mice.

## CONFLICT OF INTEREST

The author has declared that no conflict of interest exists.
